# A Transdiagnostic Perspective on Social Anhedonia

**DOI:** 10.3389/fpsyt.2019.00216

**Published:** 2019-04-24

**Authors:** Emma Barkus, Johanna C. Badcock

**Affiliations:** ^1^Cognitive Basis of Atypical Behaviour Initiative (CBABi), School of Psychology, University of Wollongong, Wollongong, NSW, Australia; ^2^Centre for Clinical Research in Neuropsychiatry (CCRN), Division of Psychiatry, Faculty of Health and Medical Sciences, The University of Western Australia, Perth, WA, Australia

**Keywords:** schizotypy, schizophrenia, eating disorders, autism spectrum disorders, posttraumatic stress disorder, depression

## Abstract

Humans are highly social beings, yet people with social anhedonia experience reduced interest in or reward from social situations. Social anhedonia is a key facet of schizotypal personality, an important symptom of schizophrenia, and increasingly recognized as an important feature in a range of other psychological disorders. However, to date, there has been little examination of the similarities and differences in social anhedonia across diagnostic borders. Here, our goal was to conduct a selective review of social anhedonia in different psychological and life course contexts, including the psychosis continuum, depressive disorder, posttraumatic stress disorder, eating disorders, and autism spectrum disorders, along with developmental and neurobiological factors. Current evidence suggests that the nature and expression of social anhedonia vary across psychological disorders with some groups showing deficient learning about, enjoyment from, and anticipation of the pleasurable aspects of social interactions, while for others, some of these components appear to remain intact. However, study designs and methodologies are diverse, the roles of developmental and neurobiological factors are not routinely considered, and direct comparisons between diagnostic groups are rare—which prevents a more nuanced understanding of the underlying mechanisms involved. Future studies, parsing the wanting, liking, and learning components of social reward, will help to fill gaps in the current knowledge base. Consistent across disorders is diminished pleasure from social situations, subsequent withdrawal, and poorer social functioning in those who express social anhedonia. Nonetheless, feelings of loneliness often remain, which suggests the need for social connection is not entirely absent. Adolescence is a particularly important period of social and neural development and may provide a valuable window on the developmental origins of social anhedonia. Adaptive social functioning is key to recovery from mental health disorders; therefore, understanding the intricacies of social anhedonia will help to inform treatment and prevention strategies for a range of diagnostic categories.

## Introduction

The need to belong is fundamental to human behavior, shaping our motivation for and maintenance of social relationships (1, 2). Understanding the nature of this need is central to appreciating how social connection buffers mental well-being (3). Instances where individuals exhibit a reduced interest in and/or pleasure from social engagement therefore present a conundrum. Accordingly, social anhedonia, a trait-like disinterest in and lack of reward from social engagement (4–7), is attracting a resurgence of interest (8). While social anhedonia is associated with social withdrawal, it is distinct from social anxiety, where withdrawal from social situations is driven by a fear of negative evaluation, rather than lack of reward from social situations. That is, social anhedonia stems from diminished positive affect from social contact, rather than heightened negative affect (9). Similarly, social anhedonia is not simply an extreme form of introversion, which is characterized by low positive emotion and a preference for solitude (10). Rather, it seems comparable to (low) social closeness captured in the personality literature (11). In short, social anhedonia is not merely a reflection of diminished social interest, but at its core entails a reduced positive appraisal of all aspects of interpersonal relationships. Although social anhedonia is a key element of schizotypy and schizophrenia (6, 12, 13), it is increasingly recognized transdiagnostically (14), spurring the development of a wide range of subjective (e.g., **Table 1**) and objective tools for its assessment.

**Table 1:** Common self-report and interview measures for social anhedonia (SA) and related constructs.

References	Name of measure	Description
Eckblad et al.^a^	Revised SA Scale (RSAS)	Self-report—trait. Captures associability, lack of social enjoyment, indifference to others.
Foulkes et al. (15)	Social Reward Questionnaire	Self-report—captures individual differences in the value of social rewards.
Fawcett et al. (16)	Fawcett–Clark Pleasure Capacity Scale (FCPS)	Self-report—people’s subjective responses to normally pleasurable situations.
Gard et al. (17)	Temporal Experiences of Pleasure Scale (TEPS)	Self-report—trait. Anticipatory and consummatory pleasure experiences.
Gooding and Pflum (18)	Anticipatory and Consummatory Interpersonal Pleasure Scale (ACIPS)	Self-report—trait. Forward looking and immediate enjoyment of social interactions.
Gooding et al. (19)	Anticipatory and Consummatory Interpersonal Pleasure Scale (ACIPS-A)	As above but for adolescents.
Kirkpatrick et al. (20)	Brief Negative Symptom Scale (BNSS)	Interview—symptom. Subscales anhedonia, intensity of pleasure and frequency of pleasure correlate with other SA scales.
Snaith et al. (21)	Snaith–Hamilton Pleasure Scale (SHAPS)	Self-report—hedonic capacity.

a Eckblad ML, Chapman LJ, Chapman JP, Mishlove M. (1982). The revised social anhedonia scales. (Available from LJ. Chapman, Department of Psychology, 1202 West Johnson Street, University of Wisconsin, Madison, WI 53706.)

The scales used in the social anhedonia literature move from the focused RSAS to the measures of social reward and the experience of pleasure in general, with a lesser or greater emphasis on the social context. These scales therefore provide different information about social anhedonia directly or the experiences that underpin it, such as social reward. Criticisms with the content of early self-report scales (22) have driven more recent efforts to uncover the multidimensional nature of social anhedonia, in part to reflect a more nuanced understanding of the cognitive processes that could be driving social anhedonia. While our understanding of anhedonia has been slow to evolve, experimental and preclinical work has provided increments in knowledge concerning hedonic experiences. Current evidence suggests that, cognitively, pleasurable or rewarding experiences, in general, comprise a “looking forward to” anticipatory component, which is cognitively driven (i.e., “wanting”) (23, 24), and an “in-the-moment enjoyment” (i.e., “liking”) component (25, 26). A third component, “learning,” ensures that individuals pair their social experiences with positive pleasurable emotions and consequently seek out similar future experiences (27). The interest in the pleasure cycle has led some authors to increasingly suggest that the broader concept of anhedonia should distinguish between motivation or seeking behaviors and in-the-moment pleasure of an experience (28). Self-report measures such as the TEPS and the ACIPS have attempted to parse these anticipatory and consummatory components of pleasure into more human social domains, with varying success. The ACIPS, although in early days of use, may prove to be an additional general measure of social anhedonia. It is recognized that pleasure, reward, learning, anhedonia, and social anhedonia are all distinct concepts. However, accumulating evidence from preclinical, translational, basic, and applied human research points to these concepts being related to one another. Processes related to the experience of pleasure and reward likely provide the mechanisms underpinning the behavioral expression of social anhedonia. Consequently, there is a need to understand specific types of anhedonia (29), which drives the encompassing nature of the current review. Understanding which components of cognitive processing are altered in people with social anhedonia—regardless of diagnostic category—could assist in improving social functioning (30, 31) and therapeutic outcomes (32). Yet, despite the potential significance of social anhedonia in both clinical and nonclinical groups, a synthesis of current evidence is lacking. Consequently, here we provide a selective review of the literature, highlighting similarities and differences in social anhedonia across a variety of presentations. Studies have been included on the basis that they have a focus on social anhedonia or anticipatory and consummatory components of reward and hedonic responses.

## Psychosis Continuum

By far, the largest body of work concerning social anhedonia relates to the psychosis continuum. Though subject to much debate, the continuum model posits that psychotic disorders comprise an extended phenotype of risk, with diagnosable disorders such as schizophrenia at one extreme end and nonclinical high, average, and low levels of schizotypy (associated with decreasing levels of psychosis risk) at the other (33). Underlying schizotypy is a multidimensional structure of personality traits broadly similar to the symptom presentation observed in psychotic disorders, lending further support to structural continuity between clinical and nonclinical experiences [see also Ref. (34)]. In the psychosis continuum literature, social anhedonia is a feature of the negative dimension and is thought to be trait-like, enduring and stable over the illness course irrespective of symptom fluctuation (5, 35).

### Nonclinical Populations and Social Anhedonia

First, we will consider studies involving nonclinical individuals expressing social anhedonia. People drawn from the general healthy community with high levels of social anhedonia report fewer personal relationships (30, 36), are less likely to be in intimate relationships (37), prefer to be alone (30), are less committed to, and experience fewer positive feelings toward, their partner when in a relationship (31). Llerena et al. (38) reported that self-reported social anhedonia was associated with less willingness to engage in social interactions, reduced social skills, and less positive responses to an experimental social interaction. Nonetheless, high levels of social anhedonia—at least in healthy young adults—are associated with high levels of loneliness (39). This suggests that while the enjoyment of, or wanting for, social interaction in people with social anhedonia is low, or subjectively dampened, it is not entirely absent, since reduction below the desired level still triggers a sense of absence and the pain of loneliness.

Neurocognitive deficits are present in some healthy individuals who express social anhedonia, although these cognitive deficits do not appear to be related to general (psychological, social, or occupational) functioning (40). Cohen et al. (40) reported that those scoring highly on social anhedonia had poorer performance on a standard assessment of attentional vigilance; similarly, Tully et al. (41) found that attentional control mediated the relationship between social anhedonia and social functioning. Although tentative, these two papers suggest that the capacity to attend to, and ultimately learn from, important aspects of one’s environment may be a key issue in social anhedonia. The results from Pflum and Gooding (42) support this idea, since participants scoring highly on social anhedonia were less able to use social context and therefore had poorer performance when it was relevant in tasks assessing social cognition. In addition, improving working memory capacity in those with social anhedonia from the general population has been reported to improve the anticipatory component of reward and lead to upregulation of brain regions related to monitoring and reward (43, 44). Within the general population, social anhedonia is associated with negative social outcomes, indicating poor engagement, maintenance, and affiliation for interpersonal relationships (45), and is predictive of the risk of developing schizophrenia in the future (13, 45).

### Psychosis Clinical Samples

Moving into clinical samples, a reciprocal relationship appears to exist between poor premorbid social adjustment and increased levels of social anhedonia in help-seeking youth at clinical high risk for psychosis (46). Potentially, this could initiate a downward spiral of loss of social skills, decreased motivation to engage, and reduced pleasure from social situations. Using an experimental social interaction paradigm, McCarthy et al. (47) reported that in patients with schizophrenia, higher ratings on social anhedonia were associated with reduced feelings of interpersonal closeness and less willingness to interact, with subjective responses during interactions with the experimental partner being related to social functioning in general. Conversely, in treatment-resistant patients with schizophrenia, *lower* levels of anhedonia–asociality predicted a longer time living in the community between admissions (48). Patients with schizophrenia and schizoaffective disorder who had high levels of social anhedonia had higher levels of symptomatology and lower levels of self-esteem, self-efficacy, subjective recovery, social support, and poorer quality of life compared to patients with intact hedonic responses and an intermediary group (49). Collectively, these studies suggest that increased social anhedonia and related constructs diminish social functioning and willingness to engage in social interactions in those at risk for or diagnosed with psychosis. Given the similar findings in nonclinical samples, social anhedonia, rather than positive psychotic symptoms, may be responsible for decreased social engagement across the psychosis continuum.

### Cognitive Underpinnings of Social Anhedonia Along the Psychosis Continuum

There have been fewer formalized experimental paradigm studies tapping the cognitive underpinnings of social anhedonia. People from the general population who score highly on social anhedonia report difficulties in controlling the effects of emotional information on behavior (50, 51), less positive affect in response to positive pictures, videos, and words (52–54), and rated sad and neutral faces more negatively (55). In patients with schizophrenia, interest has developed in “basic” symptoms; these are subtle changes from “normal” in subjective experiences of emotions, the self, and perceptions of the world (56). The basic symptoms that capture a need to consciously reflect on usual activities, reduced social motivation and emotional meaning, and lower stress thresholds were all associated with higher social anhedonia in patients with schizophrenia (57). Together, these studies suggest that in those from the general community and patients with schizophrenia, social anhedonia is associated with alterations in the subjective experience of, and objective responding to, emotionally loaded social cues.

In other clinical disorders, there has been an increasing emphasis on parcelling out the learning, wanting, and consummatory components of pleasure. Given the evidence of dysregulation in dopamine in those with psychosis, there has been much characterization of the motivational and reward-related problems reported in patients with schizophrenia (58). For example, in two studies (one using experience sampling methodology and the second employing cross-sectional questionnaires), Gard et al. (59) reported that patients with schizophrenia had intact in-the-moment (i.e., consummatory) positive emotions in response to events; however, they displayed deficits in positively anticipating future events when compared to healthy controls. However, it is only relatively recently that separating consummatory and anticipatory reward has been extended to social anhedonia across the psychosis continuum. Intact positive emotional responses to external stimuli, in those with social anhedonia, have not been consistently reported (52).

When examining the learning component, during reward paradigms, people expressing higher levels of social anhedonia changed their responding style less in reaction to social, but not monetary, rewards (60, 61), demonstrating less favorable responses to social cues than would be expected. Therefore, it appears that diminished experience of reward in social anhedonia along the psychosis continuum may be specific to social stimuli (61). Patients with schizophrenia who have social anhedonia are less able to modulate positive emotions (63), anticipate future pleasure (59), or use previous positive experiences to motivate future behaviors (63). Again, however, anhedonia in psychotic disorders is associated with high levels of loneliness (64), suggesting that the enjoyment of social interactions is diminished in people with psychosis, but sensitivity to pain from (inadequate) social connections remains. Potentially, the feeling of loneliness could derive from the reduced hedonic response to social interactions, or this could be a precursor to the tipping point between transient and chronic loneliness. Given that a significant component of social connections extends from positive responses (65), more specifically positive connection to people, reduced pleasure from social engagement could impair an individual’s capacity to gain meaningful connections. In addition, given that social withdrawal occurs in social anhedonia, it provides a potential blockage to reinstating social connections and alleviating loneliness (66). Impaired learning will then make it hard to recouple social approach behaviors and motivation with positive emotional experiences (67). Collectively, perhaps, these studies point to a breakdown in the learning component of reward, which may underpin diminished expression of anticipatory positive emotions in relation to positive future events. In turn, a deficit in anticipatory reward could impair social approach behaviors and lead to less engagement in social activities and ultimately diminish social functioning.

## Depressive disorders

Anhedonia is a core component of depressive disorders. However, there is limited research on social anhedonia specifically. Emerging literature suggests that anhedonia may be trait-like and predictive of both risk of onset and relapse for major depression (68). Yet, in depression, some argue that social anhedonia is coupled to clinical state and improves with recovery (5). One study involving clinically active patients with depression reported that those with depression had higher levels of social anhedonia compared to individuals with schizophrenia (69). Social anhedonia may be present at clinically significant levels in approximately a third of patients with depression (70). As a continuous measure, social anhedonia is associated with depressive symptom severity (70–72) and poor treatment response (68, 73). Deficits in the reward system of those with depression have been previously considered. For instance, reduced motivation for reward is thought to be driven by deficits in the anticipation of rewards, while enjoyment of in-the-moment consummatory reward remains intact (74). However, studies concerning social anhedonia in those with depression tend to capture behavioral correlates and not the cognitive underpinnings.

Evidence that social anhedonia and depressive symptoms are related in the general population could contribute evidence toward social anhedonia being viewed as a trait-like vulnerability rather than coupled with clinical state. Anticipation of a less positive response to social situations is reported in females, from the general population, with high levels of depressive symptoms compared to those with low depressive symptoms (75). Certainly, cognitive vulnerabilities for depression such as increased self-focus tend to lead to difficulties in interpersonal relationships (76). Focus on one’s own internal states reduces engagement with the social environment, blunts in-the-moment emotional responses, and diminishes social skills. For instance, reduced emotional facial expression and less smiling (77) are reported in depression and may exacerbate social anhedonia (53, 78). These psychological characteristics could compromise cognitive capacities, reducing the cognitive resources available to engage in social situations actively. In a working memory training program for those with psychometrically defined depression, although ratings of depression decreased and hedonic experiences increased, social anhedonia remained the same pre- and posttraining (79). This study suggests, that while improvements can be gained in the anticipation of future events through cognitive remediation, it might be more difficult to change patterns of behavioral responses in general. However, the possibility that working memory training could improve anticipatory reward in those with anhedonia, in general, suggests that cognitive capacities could be a target to improve positive emotions toward social engagement and may in the longer term lead to changes in social behaviors. For instance, the absence of perceived positive social cues (through cognitive biases related to anhedonia) during interactions could reduce the rewarding properties of social interactions. For example, in patients with schizophrenia, higher ratings of social anhedonia reportedly correlate with self-depreciation, guilty ideas of reference, pathological guilt, early wakening, suicidality, and observed depression (80). Other studies suggest that social anhedonia is temporally linked to episodes of clinical depression (81). In conclusion, depression and social anhedonia may co-occur in subclinical and all clinical disorders where depression is present. Further longitudinal research is needed to investigate whether social anhedonia is a stable risk temperament for depressive disorders or present only during an active depressive episode, regardless of primary diagnosis.

## Eating Disorders

Eating disorders, such as anorexia nervosa (AN) and bulimia nervosa (BN), are characterized by disturbances in behavioral and neurobiological responses to food rewards (82). There are a number of reviews that consider the role of reward, in general, in eating disorders (83–86). It is beyond the focus of the current review to reiterate the work that others have already provided focused reviews on. However, given the focus on reward as a core concern in the nature of eating disorders, hedonic responses have consistently been separated into anticipatory and consummatory components. Anhedonia, in general, is also a focus of interest in eating disorders, though developing a single unifying framework is proving a challenge (87). For example, some argue that eating disorder patients show a diminished sense of pleasure from food; others report that hedonic impact is intact but the desire for food is impaired (88–90). Some forms of eating disorders may be associated with normal levels of wanting but low levels of liking food, while BN may be associated with an excessive wanting of food that is not followed by a typical sense of pleasure (82). Such complexity illustrates the heterogeneity in eating disorders; however, it is immediately evident that reward processes are key to clinical symptoms.

Beyond reward difficulties in relation to food, current models also recognize the importance of social and emotional difficulties in the development, maintenance, and recovery from eating disorders (91, 92). These include increased sensitivity to social rejection and alexithymia, along with poor social cognition and decreased sensitivity to social rewards (93–95). Within this complex profile, some studies have examined social anhedonia in eating disorders using self-report measures. The results showed that people with AN and BN have significantly higher scores on the Revised Social Anhedonia Scale than those without eating disorders (96–98), while those who had recovered from AN had intermediate levels of social anhedonia. Levels of social anhedonia do not differ between women with AN and BN (99), are highly correlated with illness chronicity, and significantly predict problems with social and occupational functioning (97). Evidence from behavioral studies suggests women with AN avoid looking at the faces and eyes of others and find them less rewarding compared to non-eating disorder controls (100), while at the neural level, areas of hypo- and hyperactivation have been identified in response to positive and negative social exchanges, respectively (101, 102). Together, these data suggest that reward dysfunction in people with eating disorders is not specific to food-related stimuli or body image but encompasses abnormal motivational drive and pleasure from social interactions. Whether this is a cause or a consequence of eating disorders is still unclear; however, given its potential impact on recovery, social anhedonia is now serving as a specific treatment target for psychological interventions (103). Social anhedonia has been reported to reduce following an intervention comprising cognitive remediation and emotion skills training in adults with anorexia (103, 104).

The presence of social anhedonia in eating disorders suggests that there may be overlapping mechanisms for social reward mirroring those found in other groups, such as autism spectrum disorders (ASD; see the section Autism Spectrum Conditions) and along the psychosis continuum. Indeed, elevated rates of both ASD traits and psychotic symptoms are present in patients with eating disorders (105–107). The presence of ASD traits in those with anorexia has been reported to be positively related to social anhedonia (108). Delineating the differences between diagnostic categories, eating disorder subtypes, and reward components (liking, wanting, and learning) is an important direction for future research. For example, alexithymia scores were found to be strongly associated with social anhedonia in eating disorders; thus, in contrast to ASD (109), impaired enjoyment of social interactions in eating disorders may be related to difficulties identifying and expressing feelings (96).

## Posttraumatic Stress Disorder

Like depressive disorders, anhedonia is also a central symptom of PTSD (110, 111). Greater anhedonia in PTSD has been linked to an increased risk of psychotic disorder (112, 113) and poor social functioning (114). While early research focused on fear-related processes, a growing recognition of alterations in reward processing (115, 116) led to a reconceptualization of PTSD as an imbalance between approach (reward-related) and avoidance (fear-related) systems (117–119). Consequently, as with eating disorders, PTSD research has been focused on characterizing the anticipatory and consummatory behaviors related to social anhedonia. More recently, a systematic review of self-report, behavioral, and neurobiological measures yielded a complex and mixed pattern of results for reward functioning in people with PTSD. Variation in results may reflect heterogeneity of the causes, symptoms (120), and underlying reward mechanisms (121) of posttraumatic stress. Overall, however, a tendency to both reduced consummatory *and* motivational reward was noted, especially in females with PTSD and in response to positive social stimuli compared to nonsocial stimuli, suggestive of social anhedonia (118). For example, women with PTSD report less positive affect in response to positive interpersonal (social) memories, along with lowered neural activation in ventral striatal reward networks responsive to rewarding and socially salient stimuli (122). Reduced motivation to approach socially rewarding stimuli may be particularly evident in those with PTSD when greater effort is needed to gain reward (118), suggesting reduced sensitivity in the cognitive underpinning for “wanting” social connections.

In addition to an inability to feel pleasure, people with PTSD, especially related to prolonged childhood abuse, often have a different kind of hedonic problem. Specifically, traumatized individuals report an aberrant hedonic tagging such that they experience *negative* emotional responses to usually positive events. This specific form of anhedonia, termed *negative affective interference*, reflects disruption in reward learning in the context of previously adverse social experiences (123), for example, associating positive social contact (e.g., touch) with fear and sexual abuse. Self-report data indicate that negative affective interference is positively correlated with depression and PTSD symptoms (124). However, despite the well-recognized link between early childhood trauma and depression (125), schizotypy (126), and schizophrenia (127), the role of negative affective interference has received scant attention transdiagnostically and has not been considered in relation to social anhedonia specifically.

## Autism Spectrum Conditions

Autism spectrum disorders (ASD) and schizophrenia spectrum disorders are neurodevelopmental disorders with overlapping feat ures of social dysfunction (128), reflecting both shared and distinct component processes (129, 130). Early clinical descriptions of autism (131) noted a clear lack of interest in and pleasure from social stimuli, which are also associated with autistic traits in the general population, suggesting that social anhedonia may be a stable part of the broader autism phenotype (132).

Self-report measures of negative schizotypy have been used to index anhedonia in many studies concerning those with autism (133–138). For example, Rawlings and Locarnini (133) showed that scores on the Autism Spectrum Quotient (139) (measuring autistic traits) and the Introvertive Anhedonia subscale of the O-LIFE (140) were positively correlated, indicating a substantial association between autistic traits and social anhedonia in the general community. Similarly, adults with ASD were found to have significantly higher scores on the Social Anhedonia Scale (141) compared to their peers, even when dysphoric affect was controlled (142). In these studies, it is difficult to isolate social anhedonia specifically from other negative schizotypy traits (such as social anxiety) (130) or differentiate between the liking and wanting components of anhedonia. However, studies using alternative, well-validated measures of hedonic response have produced a similar pattern of results (143). For example, adolescents with ASD reported significantly less enjoyment of social situations (as measured with the Pleasure Scale) (144) compared to typical controls, while pleasure from other sources did not differ. Similarly, higher levels of autistic traits in a nonclinical sample were associated with less enjoyment of sociability (measured with the Social Reward Questionnaire) (109), even when differences in alexithymia were considered. In comparing psychiatric referrals for young people aged between 6 and 18 years, Gadow and Garman (145) found that those with ASD had higher rates of social anhedonia, while across all young people referred to the service, social anhedonia was related to higher social skill deficits, social anxiety, depression, and psychotic symptoms. This suggests that while social anhedonia might be present to some degree as a pediatric vulnerability marker for psychopathology, it appears to be more core to the presentation of ASD. Further studies are needed to understand the distinction between social anhedonia as a general risk factor for psychopathology and its presentation in ASD.

Of note, findings appear to indicate a specific problem of social anhedonia in adolescents (143), with broader reductions in social and nonsocial pleasure in adults with ASD (132, 143). However, this difference could be due to the types of measures employed or may be related to developmental factors and merits further investigation. If social anhedonia is a core feature of the broader autism phenotype, then why is this so? One possibility is that social anhedonia arises as a consequence of theory of mind deficits in people with ASD (e.g., difficulties in understanding the mental state of others make interactions less pleasurable) (146). Alternatively, experimental studies support the idea that a primary deficit in social motivation in ASD (linked to neuronal and hormonal circuitry) leads to difficulties seeking, experiencing, and learning about rewards from social interactions (147–150), leading to subsequent abnormalities in the development of the neural pathways underpinning reward and social functioning impairments. Again, however, while the motivation for social connection may be lower, the high rates of loneliness reported by people with ASD suggest that a basic need for some social bonds remains (151).

## Genetics/Neural mechanisms and Neurodegenerative disorders

While it is important to recognize social anhedonia’s presentation in multiple clinical groups, it is essential to link social anhedonia through to its biological correlates. Understanding whether social anhedonia is underpinned by one mechanism that is consistent across all disorders or whether there are multiple pathways that can lead to the same outcome holds important treatment and etiological implications. Given that there are diverse neurological correlates underpinning the pleasure cycle (152), it may be that there are multiple biological pathways underpinning social anhedonia, which can account for its presentation in multiple disorders of distinct etiological mechanisms.

Certainly, multivariate studies from the general population have pointed to social anhedonia being genetically conferred (153, 154). Similarly, single nucleotide polymorphisms (SNPs) in genes implicated in neurodevelopment have been associated with social anhedonia along the psychosis continuum (155, 156). Building on these findings, two neurobiological pathways are thought to contribute to social anhedonia. The first emphasizes the role of dopamine in reward processes and social bonding (157). Evidence linking dopamine and social anhedonia can be drawn from genetic association studies in relatives of patients with schizophrenia (158) and dopamine D2 receptor binding availability along the psychosis continuum (159). Furthermore, in Parkinson’s disease (characterized by the breakdown of dopamine-producing neurons), consummatory anhedonia is specifically blunted (160) independently of depression (161); however, social anhedonia is yet to receive specific attention.

A competing neurobiological hypothesis highlights the role of the opioid system in human affiliation and social bonding (162). The opioid system mediates both approach and avoidance behaviors in humans and animals. The opioid system has a role in fear consolidation and, therefore, may have specific relevance for the presence of social anhedonia in PTSD in particular (163). Evidence for the role of the opioid system in social anhedonia comes from genetic knockout and pharmacological models in mice (164) and a genetic association study in humans (165). However, the dopamine and opioid systems are also known to interact to underpin social bonding behaviors (166), with the nucleus accumbens providing an area of convergence for neural pathways (167). The DISC1-Q31L mutant mouse model is characterized by both abnormalities in nucleus accumbens structure and socially anhedonic-like behaviors (168). Together, these studies point to social bonding, reward processes, and social anhedonia being biologically mediated by both opioid and dopamine pathways.

The literature concerning the functional imaging correlates of social anhedonia is worthy of a review of its own. Because of space restrictions here, an overview of key and consistent findings will be given. Abnormal activity in the ventral lateral prefrontal cortex (VLPFC) has been the focus of work from one group of researchers in two related papers considering response to expected rewards (169, 170) and positive interpersonally relevant videos in a separate independent paper (171). In addition, Hooker et al. (171) found that those scoring highly on social anhedonia scales had lower levels of activity in the VLPFC and reported lower levels of positive affect during their daily lives. In addition, during a theory of mind task, activity in the medial prefrontal cortex accounted for the relationship between social anhedonia and self-reported social functioning (8). Reduced neural activity in face processing areas has been associated with social anhedonia during an emotional discrimination task (4), and smaller amygdala volumes have been related to higher social anhedonia scores in patients with schizophrenia (172). These studies suggest that brain areas involved in social cognition are associated with social anhedonia in clinical and healthy individuals.

Other imaging studies have examined the effective connectivity between brain areas in socially anhedonic individuals. Abnormal connectivity has been reported in adolescents (173), in those with depression (174), and in healthy volunteers (8, 175, 176) who score highly on self-report measures of social anhedonia or anticipatory/consummatory anhedonia. These studies highlight the importance of connectivity between “emotional regulation” and “reward” centers within the brain. Contrastingly, Wang et al. (177) reported inverse correlations between social anhedonia and cortical thickness in the post central gyrus and left inferior parietal gyrus (after controlling for physical anhedonia) but no correlations with cortical thickness for areas involved in the reward network. At this time however, the implications of cortical thickness are poorly understood. On the other hand, Enneking et al. report that in both healthy volunteers and patients with depression, higher levels of social anhedonia were related to reduced gray matter volume in the bilateral caudate nucleus (178); these results remained controlling for other symptoms and medication status in the participants diagnosed with depression. The authors point to the role of the caudate nucleus in reward-based learning and suggest it could account for difficulties in anticipating future rewards. This paper, perhaps, begins to suggest that the same neural substrates may underpin social anhedonia in healthy individuals as in patient groups. However, it does not address why social domains would be specifically affected by alterations in the caudate nucleus.

Genetic, neurobiological, and imaging studies collectively point to the importance of emotional regulation, reward circuitry, and social anhedonia. Such studies are only beginning to differentiate the wanting, liking, and learning components of social rewards; these psychological phenomena are complex manifestations of human desire/preference and hedonic in-the-moment responses. Consequently, novel imaging paradigms will be needed to access the corresponding neural mechanisms. Certainly, imaging studies emphasize the importance of reward circuitry, the development and consolidation of which can be traced back to adolescence as a key window of sensitivity (179). Adolescence appears to represent a time when reward circuits are particularly sensitive to social context and peer experiences (180, 181). This potentially points to the necessity to take into account social experiences and affiliations during adolescence to better understand the expression of social anhedonia in adults.

## Developmental Perspective

Losing the desire or not experiencing pleasure in social interactions may have profound, negative consequences at any age. Currently, it is unclear at what age social anhedonia first presents, although notably, children at familial risk for developing psychosis present with poor social skills at all stages of development (182). Adolescence may be an especially important window to measure social anhedonia [e.g., Ref. (19)], since it is marked by considerable variability in neural, affective, and social development as well as peak vulnerability for psychopathology (183). Indeed, persistent, trait-like social anhedonia in early adolescence predicts increased severity of, and poorer recovery from, depression, above and beyond other illness features (184), while elevated levels of social anhedonia in older adolescents have been associated with an increased risk of developing schizophrenia spectrum disorders later in adulthood (6, 45). This suggests that age of first presentation of social anhedonia may have different implications for well-being. Importantly, the type of stimuli perceived to be rewarding changes throughout development. Acceptance and social inclusion by peers, for example, assume particular salience in early adolescents, while emerging adults may be more sensitive to intimacy cues, such as those from potential romantic partners (185). Conversely, various forms of social defeat have been linked to the onset of anhedonia, depression, anxiety, and schizophrenia (186–189). Together, the findings suggest that there may be normative, age-related variations in the most prominent facets of social rewards *within* adolescence that require more detailed examination (190).

At the neural level, the cortical mechanisms involved in the processing of social and nonsocial rewards follow differential trajectories with age and gender, which can give rise to an imbalance between (heightened) motivation and (diminished) cognitive control during adolescence (191). Diminished activity in the ventral striatum in response to anticipation of monetary reward has been associated with anhedonia in adolescents, and ventral striatum activity in response to reward may be moderated by exposure to early life stress (192). These studies suggest that, like in adults, the ventral striatum is important in determining response to rewards, with social context, life experience, and expression of anhedonia mediating or moderating responsiveness. Few studies have focused on social reward and social anhedonia specifically. However, Healey et al. (173) reported that social anhedonia disrupted the neural response to social rewards: higher levels of activation in the medial prefrontal cortex and increased connectivity between the medial prefrontal cortex and the nucleus accumbens in response to positive social feedback were associated with higher levels of self-reported social anhedonia in adolescents. These results remained significant when other depressive symptoms were controlled for. Therefore, it appears that social anhedonia has quite specific effects on neural responses to rewards during adolescence. Much research has focused on response to reward during adolescence with regard to risk-taking behaviors, suicidality, substance use, and depression. However, specific consideration of social anhedonia, its consequences, and its relationship to social reward is lacking. Recent improvements in assessment (at self-report, behavioral, and neurophysiological levels) are beginning to offer a more detailed understanding of how social pleasure develops and goes awry (193, 194). For example, the Anticipatory and Consummatory Interpersonal Pleasure Scale for Adolescents (ACIPS-A) (19) measures the discrete wanting and liking component of reward and may help classify adolescents in the general community who are/are not at risk (e.g., of psychosis) (195) and may benefit from early intervention.

## General Discussion

### Comparison Across Different Diagnostic Groups

Social anhedonia presents across multiple psychological disorders and is detectable to variable degree in the general healthy population (14). Across populations, it appears to have consequences for social enjoyment, affiliation, engagement, and functioning over and above any symptoms attributable to diagnosis. Specifically, decreased sensitivity to social rewards is documented in schizophrenia (61), eating disorders (93), PTSD (118), and autism spectrum disorders (146). Social anhedonia, specifically, may present as a trait temperament characteristic in some individuals, while for others, it may be linked to the co-occurrence of depressive symptomatology. For instance, in patients with schizophrenia and bipolar disorder, depression (rather than cognitive functioning) has been reported to predict social anhedonia (196). The wider presentation of anhedonia also needs to be considered as a backdrop for social anhedonia. In some disorders (i.e., eating disorders), anhedonia is present but generalized across both social and nonsocial domains, while for others, such as PTSD, anhedonia may be relatively specific to social environments and interactions. Studies are also required comparing differing levels of social anhedonia across different diagnostic groups. Currently, some studies report that patients with active depression score higher than patients with schizophrenia on social anhedonia (69), while others report that patients with schizophrenia score higher than those with bipolar disorder, who both score higher than healthy controls (196). Across all studies, patient groups, regardless of diagnosis, report higher levels of social anhedonia compared to healthy controls, although it needs to be noted that the psychosis continuum work highlights wide variation in social anhedonia scores in healthy volunteer samples. Fine-grained consideration of anticipatory and consummatory components also needs consistent consideration across diagnostic groups. Broadly speaking, the psychosis continuum seems to be characterized by deficits in anticipatory but not consummatory systems (although not consistently) (59), while PTSD (121), depression (74), and ASD reflect difficulties in both and the picture for eating disorders appears to be complex with inconsistent findings reflecting symptom and etiological heterogeneity. In increasing our understanding of social anhedonia it is important to understand whether its presentation differs when it occurs in the presence of broader anhedonia compared to the specific subtype of social contexts, cues, and environments. Indeed, social anhedonia is complex: it is defined by and captures both observable social behaviors and subjective hedonic responses.

Although the exploration of anticipatory and consummatory reward begins to explicate the complex nature of reward in psychiatric conditions, we need to specifically consider these in relation to social anhedonia *within and between* different groups. The processing of reward is related to social anhedonia; however, the experience of and processes underpinning reward are not interchangeable with social anhedonia. Within each diagnostic group (and indeed in healthy populations), those who express social anhedonia are a subgroup (70) whose hedonic and reward responses likely differ from their counterparts. We must not confuse the processes underpinning social anhedonia as interchangeable with its phenotypic characterization. Stated differently, we need to better understand exactly what the mechanisms are underpinning the expression of social anhedonia and how these vary across diagnostic groups. Furthermore, there is increasing emphasis on the anticipatory and consummatory components of reward; however, the learning component of the pleasure cycle has been relatively underresearched in relation to social anhedonia.

### Psychometric and Measurement Issues

There are now a number of measures used in this area that varyingly capture social anhedonia as a trait, anticipatory and consummatory pleasure, social reward, and pleasure responses in general. While studies considering pleasure and reward provide information about the processes that underpin social anhedonia, they are not interchangeable with the use of trait measures such as the RSAS. Further psychometric work is needed to understand the shared variance between these measures. For instance, Gooding et al. (197) have reported that different aspects of personality predict scores on the ACIPS and the RSAS, suggesting subtle differences in what these two scales capture despite both being measures of social anhedonia. Furthermore, we need to begin to devise smart validity studies providing links to emotional and behavioral manifestations of social anhedonia.

There are two other, highly related, points that arise in consideration of the findings within this area. The first is the quality of studies on social anhedonia. These are largely correlational or observational in nature, regardless of whether they are considering clinical or nonclinical samples. Currently, there are no recognized clinically relevant cutoffs for most of the measures used in this field to distinguish functional versus dysfunctional levels of social anhedonia. This is particularly problematic when considering the variability in results in the psychosis continuum literature work; it may be the largest body of work on social anhedonia, but it is also the most inconsistent with its findings. Clinical cutoffs for measures would start to tease apart whether the levels of social anhedonia being expressed across different diagnostic groups, as well as between healthy volunteers and diagnostic groups, are similar in nature. In addition, a parallel issue is the necessity to begin to understand at what level social anhedonia begins to impact functioning; this, again, is equally important to healthy volunteer studies as well as clinical studies. In fact, if we can understand at what point levels of social anhedonia begin to impact social functioning in healthy volunteers, we will be able to begin to unpick whether social anhedonia or other symptomatology is driving social functioning deficits in those who do have a primary diagnosis.

The self-report nature of the scales used in research on social anhedonia also relies on the level of self-awareness that people have about their own thoughts, feelings, and behaviors in social interactions, and motivations for interpersonal communication. This is clearly problematic, particularly once we begin to consider the effects of mental health disorders on self-awareness. There is increasing evidence for subjective deficits in self (emotion) regulation in social anhedonia, while objective performance remains intact (198); these subjective deficits, however, would be sufficient to bias the processing of social information. In addition, low pleasure beliefs are documented in relation to social anhedonia (199), and these low pleasure beliefs appear to play a role in predicting the likelihood that future pleasurable events will occur (200). These studies point to there being cognitive biases related to social anhedonia, which could influence the likelihood of similarly biased responding on social report scales. The possibility that biases could shape responses also goes back to the paradoxical relationship between social anhedonia and loneliness and the number of studies that demonstrated those with social anhedonia *do* engage in significant social relationships: If those with social anhedonia do not gain pleasure or reward from social interactions, then why do they appear to experience negative consequences from the absence of affiliative experiences? Perhaps, we are currently missing important information about the processing of social interactions and the subjective gain from them by relying on predominantly self-report data within the area of social anhedonia.

There are some innovative studies within this area, however, that do attempt to address the potential for poor social awareness in social situations in those who express social anhedonia. A series of studies by Blanchard and colleagues (38, 47, 53, 65) uses a paradigm whereby participants interact with an experimenter who rates their social interaction as well as the participant rating the social interaction. This permits the marrying of perceptions of social engagement and interaction style with ratings reflecting the subjective and (more) objective experience ratings. More studies are needed that make use of behavioral indicators and measures of social behaviors for social anhedonia alongside reliable and valid self-report scales.

Interestingly, a number of more recently published studies are using the term “negative schizotypy” to capture social anhedonia. Negative schizotypy is a broader construct than merely social anhedonia and also encapsulates social anxiety, flattened affect, and anhedonia in general. Therefore, care needs to be taken in a consistent use of terminology so that it is clear from the outset what constructs have been measured to ensure that the significance of the results can be clearly appreciated. Furthermore, social anhedonia needs consideration as a distinct form of anhedonia—with separable neural substrates (201). When considering the clinical end point of schizophrenia, work is emerging that anhedonia is a distinct feature of negative symptoms worthy of consideration (202). Indeed, future research needs to consider whether social anhedonia is distinct from other symptoms of psychiatric diagnoses. Similarly, recent studies have examined whether social anhedonia is a separable construct from other schizotypal traits. Support for this proposal comes from recent psychometric work, which revealed that negative affect and social anhedonia load on separate dimensions of the broader schizotypy construct (203), while other works suggest that social anhedonia loads onto both positive and negative features of schizotypy (204, 205). Therefore, further empirical evidence is needed to better understand the relation of social anhedonia to other schizotypal traits and to other mental health disorders considered here.

### Future Directions and Conclusions

Social interactions are the most complex and ambiguous environments human beings place themselves in. Many explicit and implicit “rules” govern what is considered appropriate and what can be expected from any one interaction. Consequently, they are settings rife for miscommunication and misunderstanding. It requires intricate examination to determine exactly what the rewarding components of social interactions are. Social interactions are highly dynamic, complex circumstances that seem more inclined to produce anxiety than lead to rewards given their ambiguities. For those with social anhedonia, whose sensitivity to the positive gains from social interactions may be limited, it is easy to see why relationships might be considered best avoided. Individuals with social anhedonia may require larger perceived gains before they engage in social approach behaviors. We currently have a poor understanding of how social anhedonia relates to social motivation [e.g., Ref. (38)]. However, studies point to those with social anhedonia seeking out personal relationships, albeit with reduced positive experiences (31); therefore, those with social anhedonia have *some *motivation for belongingness and are aware when belongingness needs are unmet (i.e., they *feel* lonely). A greater understanding of the perceived rewards from social interactions and interpersonal relationships would assist in determining what motivates people to make connections with one another. It is becoming particularly important to understand this given the emerging body of work demonstrating the detrimental effects of loneliness and social disconnection on physical and mental health (3, 64, 206).

Currently, there is limited understanding of the neurodevelopmental origins of social anhedonia or the differences in social anhedonia experienced earlier (e.g., as a personality trait) versus later in life (e.g., in the context of adult-onset disorder). The neurobiological and neural correlates implicated in emotional regulation and reward provide likely key biological pathways for social anhedonia. Adolescence represents a key window of vulnerability given that reward processing and social needs undergo maturational changes (207, 208). The psychological (e.g., temperament, mood), learning (e.g., stresses, life e vents, trauma, attachment), and cognitive (e.g., anticipatory and consummatory reward) correlates of social anhedonia require exploration in children and adolescents. The socialization of reward processes may represent a point of psychological vulnerability (209), particularly how children learn to express and receive reward from social interactions. Understanding how reward from social interactions is gained will not only assist in understanding the development of social anhedonia but may also begin to elucidate the reason social connections buffer physical and mental health (210).

## Author Contributions

EB and JB both conducted the literature searches that generated the papers to be included. Both authors contributed to the text within the manuscript and edited different versions. EB integrated the manuscript to ensure consistency in approach and style of writing.

## Conflict of Interest Statement

The authors declare that the research was conducted in the absence of any commercial or financial relationships that could be construed as a potential conflict of interest.
